# Efficacy of Mouthwashes Containing Hydrogen Peroxide on Tooth Whitening

**DOI:** 10.1155/2015/961403

**Published:** 2015-07-30

**Authors:** Muhammet Karadas, Omer Hatipoglu

**Affiliations:** Department of Restorative Dentistry, Faculty of Dentistry, Recep Tayyip Erdogan University, 53100 Rize, Turkey

## Abstract

The aim of this study was to analyze the efficacy of mouthwashes containing hydrogen peroxide compared with 10% carbamide peroxide (CP) gel. Fifty enamel-dentin samples were obtained from bovine incisors and then stained in a tea solution. The stained samples were randomly divided into five groups according to the whitening product applied (*n* = 10): AS: no whitening (negative control), with the samples stored in artificial saliva; CR: Crest 3D White mouthwash; LS: Listerine Whitening mouthwash; SC: Scope White mouthwash; and OP group: 10% CP Opalescence PF (positive control). Color measurements were carried out with a spectrophotometer before staining, after staining, and on the 7th, 28th, and 56th day of the whitening period. The data were analyzed using two-way analysis of variance followed by a Tukey post hoc test. The color change (Δ*E*) was significantly greater in all the groups compared to that of the AS group. After 56 days, no significant differences were found among the mouthwash products with respect to color change (*P* > 0.05). The whiteness of the teeth treated with the mouthwashes increased significantly over time. Nevertheless, the color change achieved with the mouthwashes was significantly lower than that achieved with the 10% CP at-home bleaching gel.

## 1. Introduction

Patients today demand more than a healthy mouth and a perfect smile. The color and aesthetics of teeth are very important to patients, as they influence self-esteem and professional relationships [1]. Tooth bleaching has become one of the most popular and common esthetic dental procedures for whitening discolored teeth in modern esthetic dentistry. This process is a relatively simple and conservative option compared to other forms of treatment, such as veneers and crowns [2]. Tooth bleaching refers to any procedure that does not use restorative materials and that changes the color and appearance of teeth that were discolored due to intrinsic and extrinsic staining [3]. Fundamental vital tooth bleaching techniques can be generally classified as at-home (dentist-supervised nightguard bleaching), in-office or power bleaching (professionally administered) and over-the-counter (OTC) or mass-market products [4–6].

OTC products are a low-cost alternative for white discolored teeth without dentist supervision [7, 8]. Different OTC agents are available in supermarkets and pharmacies and on many websites [8]. These products generally contain lower levels of a whitening agent and are self-applied to teeth by means of gum shields, strips, paint-on brushes, toothpastes, and mouthwash products. They commonly require two daily applications for up to 2 weeks [6].

Mouthwashes are very popular oral hygiene agents. They act to chemically control cariogenic biofilms and have remineralizing therapeutic properties. Due to the increased concern of patients' in recent years about dental esthetics, the number of mouthwash products containing hydrogen peroxide (HP) has risen significantly [9]. HP penetrates the tooth and produces free radicals, which attack and break apart the chromophore bonds of large, long chain, dark-colored molecules; this eventually breaks down the molecules and chromophore bonds, resulting in changes in tooth color [6]. However, in some cases, HP may not whiten teeth substantially due to the method of application and the length of time it is in contact with the teeth [3].

A few studies have evaluated the effectiveness of mouthwashes and whitening agents. Despite the increased number and sales of whitening products and mouthwashes, there is little evidence of their effectiveness. The tooth whitening of mouthwashes may also differ, depending on the constituents of the product. Therefore, the purpose of this study was to evaluate at different periods of immersion the whitening effect of three mouthwashes containing HP compared with the whitening effect of 10% carbamide peroxide (CP) used in at-home tray bleaching gels. The null hypotheses were that (1) the mouthwashes would not have any effect on the color change of teeth, (2) the immersion time in the mouthwashes would not influence tooth-whitening results, and (3) there would be no significant differences among the mouthwashes used.

## 2. Materials and Methods

### 2.1. Preparation of the Samples

Fifty extracted bovine incisors were selected for this study and cleaned with a periodontal hand scaler. They were stored in 0.5% chloramine-T solution and used within 2 weeks of extraction. Teeth with spots and fractures were excluded from the study. Enamel-dentin sections (dimension, 5 × 5 mm; thickness, 3 mm) were obtained from the midcoronal regions of teeth using water-cooled diamond disks (Impect PC10; Equilam Lab Equip, Diadema, SP, Brazil). The dentin surfaces were polished to standardize the thickness of each sample. Using molds, each enamel-dentin sample was individually mounted in transparent acrylic resin to expose the enamel surface. Each sample was polished for 10 s across the buccal surface with the use of a prophylaxis paste, applied with a polishing brush under manual pressure at a low-speed contra-angle. Then, each sample was washed with distilled water for 10 s. The prepared samples were immersed for 7 days in a tea mixture to allow the bleaching effectiveness of the 4 products to be compared on a set of stained samples. The tea solution was prepared by brewing 3.5 g of black tea in 100 mL of boiling distilled water for 10 min (Çaykur, Altınbaş Tea, Rize, Turkey). Then, the samples were washed in distilled water for 60 s.

### 2.2. Whitening or Bleaching Procedure

The stained samples were randomly assigned to five groups (*n* = 10) as follows: an AS group (negative control): the samples were immersed in artificial saliva; a CR group: the samples were immersed at 37°C in 30 mL of a whitening mouthwash (Crest 3D White) for 4 min daily [10] for 56 days; an LS group: the procedure was the same as that in the CR group but with a different whitening mouthwash (Listerine Whitening); an SC group: the procedure was the same as that in the CR group but with a different whitening mouthwash (Scope White); and an OP group (positive control): a 1.5–2 mm layer of bleaching gel (Opalescence PF 10% CP) was spread on the enamel surface for 4 h daily for 14 days. The samples were immersed at 37°C in artificial saliva [11] for the rest of the day. The constituents of the mouthwashes and those of the bleaching gel used in this study are presented in Table 1.

### 2.3. Color Evaluation

Before color measurement, the samples were dried with absorbent paper. A trained examiner conducted the color measurements of each sample against a white background in standardized D65 daylight using a digital spectrophotometer (VITA Easyshade Advance, Zahnfabrik, Bad Säckingen, Germany). The spectrophotometer was calibrated according to the manufacturer's instructions by using the calibration plate. The following spectrophotometric data were recorded for each sample: *L* ^*∗*^, *a* ^*∗*^, and *b* ^*∗*^ coordinate values created by the Commission Internationale de l'Éclairage. The *L* ^*∗*^ value represents the degree of lightness in a sample and varies from black (0) to white (100). The *a* ^*∗*^ and *b* ^*∗*^ values represent the degree of red (+*a* ^*∗*^)–green (−*a* ^*∗*^), yellow (+*b* ^*∗*^)–blue (−*b* ^*∗*^) in the samples, respectively. Each sample color was measured at 5 time points: at baseline; after staining; and at 7 days (time 1), 28 days (time 2), and 56 days (time 3) after immersion in the respective mouthwashes. The difference between the two colors was calculated by the following formula: Δ*E* = [(Δ*L* ^*∗*^)^2^ + (Δ*a* ^*∗*^)^2^ + (Δ*b* ^*∗*^)^2^]^0.5^.

Color differences after staining were calculated using baseline color parameters. The Δ*E*, Δ*L*, Δ*a*, and Δ*b* values after 7, 28, and 56 days were calculated using color parameters after staining. In the OP group, these parameters were calculated at 7, 14 (recorded as 28 days in this study), and 56 days of the treatment period.

### 2.4. Statistical Analysis

PASW Statistics software 18 (SPSS Inc., Chicago, IL, USA) was used to analyze data. The data obtained after staining were analyzed with a one-way ANOVA to prevent possible differences in color among the groups. After the whitening process, the color parameters (Δ*E*, Δ*L*, Δ*a*, and Δ*b*) were analyzed with a two-way ANOVA (mouthwashes and time). Multigroup comparisons were conducted with the Tukey test at a 95% confidence interval.

## 3. Results

The mean values of the color parameters for each group after staining are given in Table 2. After 7 days of immersion in the tea solution, *L* ^*∗*^ values decreased from baseline recordings, whereas *a* ^*∗*^ and *b* ^*∗*^ values increased. One-way ANOVA revealed no significant difference among groups for each color parameter (*P* > 0.05).

The means and standard deviations of the Δ*E* values after whitening are shown in Table 3. At the end of the whitening process, there was no statistical difference in the color change (Δ*E*) among the mouthwash groups (*P* > 0.05). The color change (Δ*E*) in the OP group was significantly higher than those in the other groups. The results of the two-way ANOVA showed that the immersion time and mouthwashes (groups) and their interaction had a significant effect on the color change (Δ*E*) (*P* < 0.01). The Δ*E* values of all the groups were significantly different than the Δ*E* value of the AS group at 7, 28, and 56 days.

**Figure 1:**
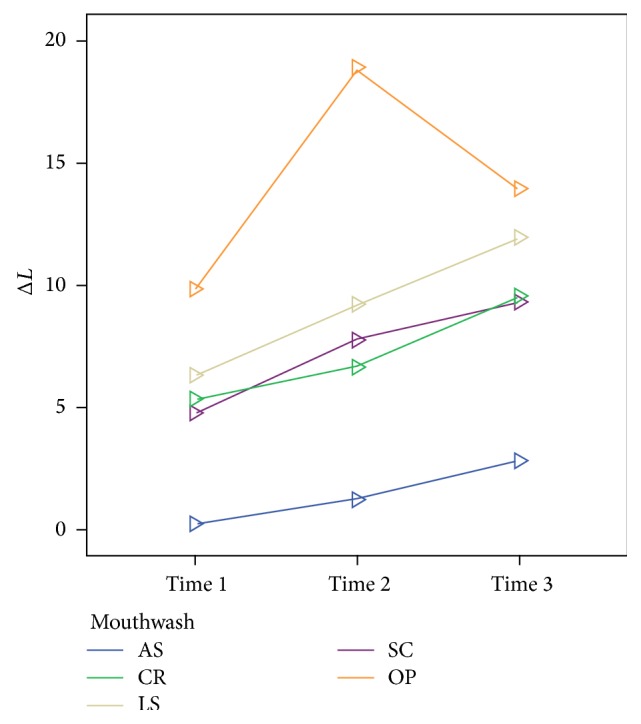
Mean Δ*L* values after whitening at 7, 28, and 56 days.

**Figure 2:**
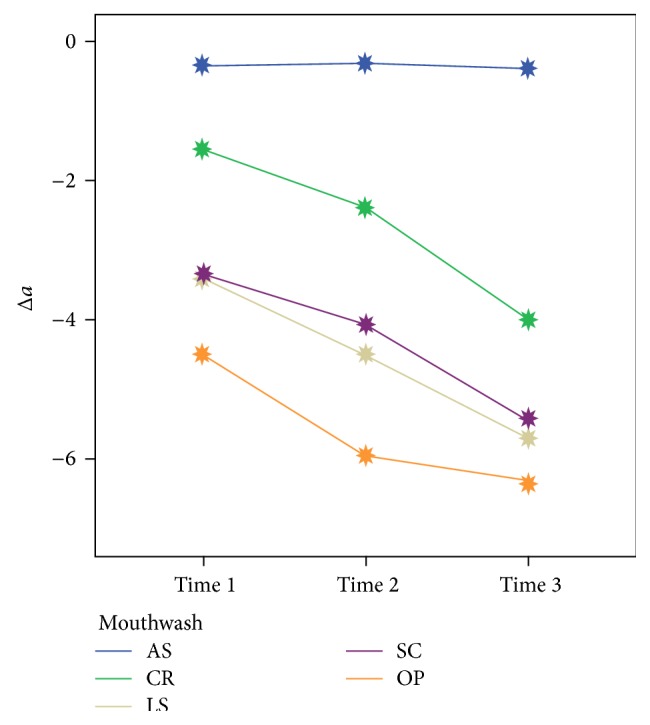
Mean Δ*a* values after whitening at 7, 28, and 56 days.

**Figure 3:**
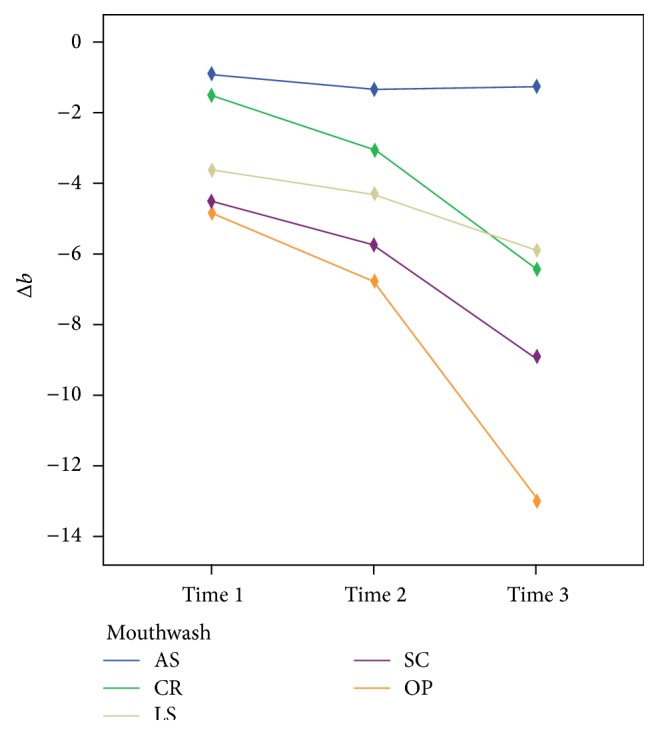
Mean Δ*b* values after whitening at 7, 28, and 56 days.

The means of the Δ*L*, Δ*a*, and Δ*b* values after whitening are shown in Figures 1, 2, and 3, respectively. In all groups, except for the AS group, the Δ*L*, Δ*a*, and Δ*b* values changed significantly over time (*P* < 0.05). After the 56-day treatment period, no significant difference for the Δ*a* values was found among the whitening products (*P* = 0.16), but the Δ*L* and Δ*b* values were significantly different (*P* < 0.05). Δ*L* values decreased in the OP group after 14 (28 in Table 2) days, whereas they increased in the other groups over time. The redness and yellowness of all the samples decreased over time.

## 4. Discussion

This in vitro study evaluated color changes of stained teeth treated with commercially available mouthwashes containing HP compared with those of teeth whitened in a 10% CP gel. The findings of the two-way ANOVA revealed that the immersion period and the mouthwashes had a major influence on color changes. Thus, the null hypothesis that the mouthwashes would have no effect on the whitening of stained teeth was rejected.

One of the most prevalent drinks in the world is tea, following water. The reported health benefits of tea have made the beverage increasingly popular, but patients are concerned about its effects of staining on teeth [12]. In vitro studies have demonstrated the staining effects of coffee, red wine, and tea [13, 14]. In the present study, tea staining was preferred because tea has been proven to have a higher capacity for staining teeth than other solutions, such as coffee or chlorhexidine [15, 16].

The surfaces of the samples were not flattened before the experiment in order to simulate clinical situations. This situation might have led to greater variations among the samples in the adsorption of color molecules and measurement of color because of irregularities in the surface textures of the samples [17].

As reported in previous studies, bovine incisors were selected to assess the tooth color change because of the ease of standardization and obtaining samples [17, 18]. The use of human teeth in in vitro investigations is limited due to ethical restrictions [19]. Extracted human teeth generally have restorations or caries that interfere with the color analysis of teeth. On the other hand, bovine teeth provide an adequate flat surface, making it easier to obtain standardized measurements [20]. As the chemical composition and structure of bovine teeth are similar to those of human teeth, bovine tooth hard tissues are often used as substitutes for human teeth in research [21]. A previous study reported that the staining of bovine and human teeth was similar, as were the effects of whitening [18].

Whitening mouthwashes have a low concentration of HP and sodium hexametaphosphate, potassium pyrophosphate, and sodium citrate. These ingredients work to whiten teeth either by bleaching or by removal and control of stains. Hydrogen peroxide diffuses through the organic matrix of tooth and produces free radicals that lead to successful whitening [22, 23]. However, the efficacy of whitening mouthwashes may be decreased by the fact that they are in contact with the teeth for a short period of time compared with bleaching gel for use at home. The results of this study showed that the amount of time the stained teeth were immersed in the mouthwash was a significant factor for tooth whitening.

Sodium hexametaphosphate has multiple binding sites and antitartar properties that help prevent staining of teeth. Also, known as polypyrophosphate, sodium hexametaphosphate chemically removes existing stains and provides long-lasting inhibition of new-stain chromogen adsorption to the tooth surface [24]. In the present study, mouthwashes containing sodium hexametaphosphate did not have an effect with respect to color change that was statistically significant compared to the other tested mouthwashes.

The literature is somewhat contradictory with respect to the effectiveness of whitening mouthwashes. A previous study reported that different peroxide-based whitening mouthwashes did not have a bleaching effect on stained teeth after a 21-day application period [25]. On the other hand, Torres and colleagues reported that the color change achieved with whitening mouthwashes used for 12 weeks was similar to that achieved with 10% CP used for 14 days [26]. In a recent study, de Jaime and colleagues examined the efficacy of a mouthwash containing HP compared with 10% CP and reported that one mouthwash (Colgate Plax Whitening) was able to whiten stained enamel, but they reported that the amount of color change was significantly lower than that obtained with 10% CP used for 28 days [10]. In the present study, the color changes (Δ*E*) of all the samples treated with the mouthwashes were significantly lower than those of the samples treated with the 10% CP at all of the evaluated time intervals, but they were significantly higher than those of the negative control group. In this study, all of the mouthwashes were used according to the recommendations of their manufacturers. Comparing this study to other studies is challenging due to a number of possible factors, including study protocol differences, staining level of samples, sample preparation, mouthwash application protocol, and in vivo and in vitro conditions.

Home bleaching treatment caused teeth whitening that was significant compared with the whitening caused by use of the mouthwashes. This difference often depended on the changes of Δ*L* and Δ*b* parameters obtained with home bleaching gel, which were statistically significant compared to the mouthwashes. For Δ*a*, there were no significant differences among the whitening products at the end of treatment period. However, the Δ*L* values of the OP group decreased after 14 days; this reduction was likely caused by color regression that the organic substances of the artificial saliva might contribute to [27]. Li and colleagues reported that most of the color regression was stimulated by the *L* ^*∗*^ value [28]. Despite completion of the home bleaching treatment, the Δ*a* and Δ*b* values continued to decline, which may be explained by the presence of the remaining oxygen radicals in tooth structure or some alterations within the tissues of the teeth. The changes in the color parameters of tested mouthwashes were statistically similar after the completion of whitening treatment.

The 10% CP was chosen as the positive control group because previous studies confirmed that it was a safe and effective technique; this has been reported in the majority of publications on home bleaching in the last 20 years [29]. A previous study also reported that the most acceptable way to whiten teeth was the at-home bleaching method [30]. Research has demonstrated that the whitening effect achieved by home bleaching was stable and long lasting [31]. Home bleaching gel (10% CP) contains 3.5% HP, and this percentage is greater than that found in the mouthwashes examined in this study. Although home bleaching HP/CP gel in a tray has limited contact with teeth and gums, mouthwash is in contact with all the oral mucosa [26].

## 5. Conclusions

Within the limitations of this in vitro study, each of the tested mouthwashes increased the whiteness of teeth over time, bleaching the stained teeth. However, none of the mouthwashes were as effective as 10% CP at-home bleaching gel. No significant differences were found among the mouthwashes with respect to color changes (Δ*E*). When the samples were exposed to whitening products, the Δ*L* values and Δ*b* values showed significant differences according to the product used.

## Figures and Tables

**Table 1:** Details of mouthwashes products and bleaching gel used in this study.

Brand name (code)	Manufacturer	Material ingredient
Listerine Whitening mouthwash (LS)	Johnson & Johnson Healthcare Products, Skillman, NJ, USA	Water, alcohol (8%), hydrogen peroxide, tetrapotassium pyrophosphate, pentasodium triphosphate, citric acid, poloxamer 407, flavor, sodium saccharin, and sucralose

Scope White mouthwash (SC)	Procter & Gamble, Cincinnati, OH, USA	Water, glycerin, alcohol (5%), 1.5% hydrogen peroxide, hexametaphosphate, poloxamer 407, sodium citrate, flavor, sodium saccharin, and citric acid

Crest 3D White Multi-Care whitening mouthwash (CR)	Procter & Gamble, Cincinnati, OH, USA	Water, 1.5% hydrogen peroxide, propylene glycol, sodium hexametaphosphate, poloxamer 407, sodium citrate, flavor, sodium saccharin, and citric acid

Opalescence PF 10% (OP)	Ultradent Products Inc., South Jordan, UT, USA	Glycerin, water, xylitol, carbamide peroxide, flavor, carbomer, PEG-300, sodium hydroxide, potassium nitrate, EDTA, and sodium fluoride

**Table 2:** Means and standard deviations (SD) of color parameters after being stored in tea solution.

Group	Δ*E*	Δ*L*	Δ*a*	Δ*b*
AS	12.80 (3.01)	−11.84 (3.45)	4.54 (1.32)	7.28 (3.10)
CR	11.65 (4.12)	−8.35 (2.23)	3.37 (1.87)	5.85 (3.79)
LS	13.62 (4.56)	−9.79 (3.03)	4.14 (1.14)	8.97 (4.03)
SC	14.25 (6.10)	−10.98 (5.01)	5.30 (3.13)	6.75 (2.18)
OP	12.90 (5.10)	−10.86 (5.23)	3.40 (2.30)	5.79 (3.07)

**Table 3:** Means and standard deviations (SD) of color changes (Δ*E*) after whitening.

Group	Δ*E* (SD)		
	Time 1	Time 2	Time 3
AS	1.23 (0.45)^Aa^	1.98 (0.76)^Aa^	2.28 (1.35)^Aa^
CR	5.82 (1.43)^Ba^	7.85 (2.71)^Ba^	12.42 (4.47)^Bb^
LS	8.51 (1.45)^Ba^	11.35 (3.93)^Cb^	14.70 (3.39)^Bc^
SC	7.84 (1.72)^Ba^	10.88 (3.03)^Cb^	14.34 (4.87)^Bc^
OP	12.18 (4.37)^Ca^	21.35 (2.73)^Db^	20.28 (5.63)^Cb^

Different uppercase letters represent statistically significant difference among groups. Different lowercase letters represent statistically significant difference at the time intervals evaluated (*P* < 0.05).
